# Supplementary material for: PhenoSV: interpretable phenotype-aware model for the prioritization of genes affected by structural variants
Source: Nat Commun. 2023 Nov 28;14:7805. doi: 10.1038/s41467-023-43651-y (PMC10684511; doi:10.1038/s41467-023-43651-y)
Supplement: Supplementary file 1 — Supplementary Information [file 41467_2023_43651_MOESM1_ESM.pdf]

Supplementary Materials

Supplementary Notes

PhenoSV-light training and testing

We recognize that the most time-consuming step for executing PhenoSV involves annotating SVs, which requires querying 238 features for all genome segments impacted by SVs. To enhance the usability of PhenoSV, we developed a lightweight version of the model, aptly named PhenoSV-light. PhenoSV-light consists of 42 important features with much improved annotation efficiency and minimally compromised predictive accuracy. The selection of these 42 important features was guided by feature importance results (Figure 3e-g), which demonstrated that combining all feature categories together led to the best results, outperforming the use of individual feature categories alone. Additionally, we observed that important features differed between coding SVs and noncoding SVs. Accordingly, we selected the top 5 important features from each of the 5 categories for coding SVs and noncoding SVs, respectively, resulting in the final set of 42 important features in total (Table S5). PhenoSV-light was trained using the same training and validation datasets, and we utilized the same hyperparameters as those used during PhenoSV trainings. To test whether PhenoSV-light can be used as an effective alternative of the original PhenoSV model, we compared model performance between two models using the same test datasets in the main manuscript. Figure S2 demonstrates that PhenoSV-light achieved largely comparable prediction accuracy to PhenoSV, except for translocations. This indicates that PhenoSV-light offers a highly efficient alternative for most SV types. As a result, PhenoSV-light becomes a practical and effective option for various analyses, presenting a more accessible and time-saving approach for researchers using the PhenoSV tool.

Sensitivity analysis of window size selection for SV proxies

PhenoSV uses 100bp deletions to approximate impacts of insertions and noncoding breakpoints of inversions. To assess the influence of the window size settings on model predictions, we conducted sensitivity analysis by comparing PhenoSV predictions in the test dataset of insertions with window sizes being 50bp, 100bp, 150bp, 200bp, 300bp, and 500bp. As shown in Figure S3, PhenoSV predictions were highly correlated across different window sizes, ranging from 0.95 to 1, and the model’s overall performance achieved nearly identical AUCs. The results demonstrate the robustness and stability of PhenoSV predictions when choosing different window sizes for SV proxies.

Splitting datasets by chromosomes and splitting datasets by random

In this study, we implemented a chromosome-based splitting strategy to divide the training, validation, and hold-out test datasets. This approach ensures that SVs in validation and the hold-out sets do not overlap with SVs in the training set, which is a commonly used strategy to prevent information leakage and ensure reliability of our test results. StrVCTVRE is one of the examples that adopted this strategy, where they used leave-one-chromosome-out for cross

validation and utilized specific chromosomes from ClinVar as the hold-out test set. To assess the validity of the strategy of splitting by chromosomes, we conducted experiments using random splitting of the dataset. We kept the numbers of pathogenic SVs and benign SVs, coding SVs and noncoding SVs in the training, validation, and hold-out test datasets the same as in the chromosome-based strategy. The results, as shown in Table S6, indicated that random splitting led to improved performance in the hold-out test dataset for both coding SVs (AUC of random split: 0.948; AUC by chromosome: 0.911) and noncoding SVs (AUC of random split: 0.89; AUC by chromosome: 0.86), On the other hand, the performance in the independent test datasets for small SVs (AUC of random split: 0.876; AUC by chromosome: 0.874) and large SVs (AUC of random split: 0.769; AUC by chromosome: 0.770) remained nearly the same for both splitting strategies. Although the results indicated similar model performance metrics in the independent test datasets for both splitting approaches, it is crucial to acknowledge that random splitting may lead to inflated performance results within the hold-out test set due to the potential presence of information leakage.

Potential issues of model overfitting

Given the large feature set used in the PhenoSV model, we acknowledge the potential risks of overfitting, particularly if there are strongly correlated features. To address this concern and mitigate the overfitting issue, we employed several techniques during our analysis. First, we implemented model regularization techniques, including drop-out layers and weight decays, during the model training process. These regularization methods help prevent the model from becoming overly sensitive to the training data, reducing the risk of overfitting and enhancing its generalization. Second, we took the precaution of splitting our training, validation, and hold-out test datasets by chromosomes to avoid information leakage and maintain the integrity of the test results. Thirdly, comparable performance between PhenoSV and PhenoSV-light demonstrated that the large feature set is less of a concern in terms of leading to overfitting (**Figure S2**). Taken together, the PhenoSV model has been designed and trained with careful attention to overfitting issues.

Ambiguities of pathogenicity labels with different disease definitions

The pathogenicity assessment of SVs can exhibit variability based on disease delimitations, and even the labels within the ClinVar dataset may sometimes introduce ambiguities. Specifically, the labels include: "benign," "benign/likely benign," "likely pathogenic," "pathogenic," "pathogenic/likely pathogenic," "uncertain significance," and "conflicting interpretations of pathogenicity." To handle this issue, two strategies are worthy of considerations: treating SV classification as a multi-category task, or training disease specific models. However, PhenoSV adopted a distinct strategy by training a generic model by treating SV pathogenicity labels as a binary variable. First, treating SV pathogenicity labels as a binary variable is a commonly employed strategy in existing machine learning-based models, such as in CADD-SV, SVFX, and StrVCTVRE. The main reason is that binary classification can facilitate minimization of loss function and increase interpretability of models on a quantitative scale. Therefore, we focused our model training on distinguishing between pathogenic SVs and

benign SVs using binary labels. However, this approach allows for a straightforward interpretation and the continuous confidence scores (ranging from 0 to 1) can be used to infer the probabilities of an SV being pathogenic in general, irrespective of disease types. In the next step, when phenotype or disease information is available, we use the information to further fine-tune the score to reflect the pathogenicity of variants with respect to specific diseases. To test whether PhenoSV can keep the ordinal information of SV pathogenicity (benign, likely pathogenic, and pathogenic) even trained with binary labels, we compared the predicted PhenoSV scores over the three categories "benign", "likely pathogenic" and "pathogenic" in the hold-out test dataset. As shown in Figure S5, the median pathogenicity score predicted by PhenoSV is 0.05 for benign SVs, 0.85 for likely pathogenic SVs, and 0.91for pathogenic SVs. This increasing trend suggested the advantage of training SV pathogenicity using binary labels. Second, training a disease-specific model with combined inputs of SV features and patient's phenotype terms can be appealing, compared to our current procedure of training a general model and then fine-tune model output using phenotype terms when available. However, to implement this strategy, we encountered the practical problem that most of the training samples do not have the corresponding phenotypes or even the disease information. With reduced sample size, the results are not satisfactory even in cross validation settings. Furthermore, as there are almost 18,000 possible HPO terms, adding raw HPO terms greatly increased the model complexities even when phenotype embedding is used in the predictive model. Thus, we opt to a generic model and utilize extra genotype-phenotype associations (e.g. Phen2Gene) to infer SV-disease associations. This procedure has the advantage of working on both relatively common diseases with general disease descriptors (such as disease name only without HPO terms) and rare diseases with more specific phenotype terms (such as a list of HPO terms).

Imbalanced numbers of coding SVs and noncoding SVs for training

There exists significant class imbalance between the numbers of coding SVs and noncoding SVs in the training dataset due to the much better understanding and disease annotation of coding variants (which may directly disrupt gene products) versus noncoding variants (which may target regulatory regions that influence levels of gene expression). While we recognize this issue, we did not adopt simulation approaches to artificially generate structural variants or under/over sampling strategies, to balance the numbers of coding SVs and noncoding SVs. The main reason is that the performance of computational approaches critically depends on how the simulation is performed for noncoding variants, yet it is difficult to justify what is the appropriate simulation strategy for the pathogenicity of noncoding SVs. Instead, to alleviate the class imbalance issue, we made the coding SVs and noncoding SVs "look alike" in the input feature space. Specifically, we segmented coding SVs into sequences of noncoding and coding regions that the SVs impact directly. If we only input the noncoding regions that noncoding SVs impact directly, the coding SVs and noncoding SVs are straightforward to be distinguished by the model through features such as exon annotations. Thus, for noncoding SVs, we segmented coding and noncoding regions within a given distance or TAD (see Methods, SVs segmentation). Only masks of attention heads between coding SVs and

noncoding SVs are different. In this way, we essentially incorporated into the model the information that noncoding SVs learned from large number of coding SVs.

Interpretations of disease-associated common SVs

In this study, we filtered out common SVs from the training dataset. The rationale behind this step lies in the fact that removing common SVs can help decrease false positive rate in the training dataset. Moreover, by removing common SVs, PhenoSV is steered to capture features that predicts SV pathogenicity, rather than being confounded by the distinction between rare and common SVs. Although some disease-risk SVs are germline SVs and commonly presented in large human cohorts, we anticipate that the challenge in clinical interpretation of SVs is to identify highly penetrant variants with large effect sizes, rather than finding disease associated polymorphisms as they usually serve as proxy markers for another disease-casual genetic mutation within a linkage disequilibrium block. For example, a 32kb LCE3C/LCE3B deletion (chr1: 152583066-152615265, GRCh38) has been shown to be associated with risk of psoriasis1, and it appears in 64% psoriasis patients and 55% controls. Since this deletion was not in our training dataset, we investigated this common variant using PhenoSV. PhenoSV predicted this SV to be benign with a score of 0.009. When examining genes separately, LCE3C has a score of 0.008 while LCE3B has a score of 0.011. We then searched the genomic region of this deletion in ClinVar and found a 203kb copy number loss that covers the entire 32kb region and contains both LCE3C/LCE3B, yet this copy number loss is asserted as being benign (VCV000152664.1, chr1: 152526704-152729716, GRCh38). Therefore, this SV, which is a genetic polymorphism, may be associated with diseases with small effect sizes of OR=1.4, but a complete loss of the region does not impact disease status.

Different thresholds of PhenoSV scores in transmission analysis

To explore whether different thresholds of PhenoSV scores when defining pathogenic and benign SVs will change the conclusion in the transmission analysis, we re-conducted the analysis by assigning the top 30% SVs as pathogenic and the bottom 30% SVs as benign based on PhenoSV score quantiles. (Paternal SVs: <=0.31 as benign, >=0.58 as pathogenic, Maternal SVs: <=0.37 as benign, >=0.71 as pathogenic). We compared the original results using 0.5 as the cut-off value (**Table S7**) and the new results using quantile scores as the cut-off values (**Table S8**). We found that different thresholds do not influence the overall conclusion of the analysis. Specifically, predicted pathogenic paternal SVs exhibited over-transmission pattern to cases with the transmission rate being 0.71 (0.5 cutoff, binomial test p-value = 0.01) and 0.72 (quantile cutoff, binomial test p-value=0.02), respectively. Predicted benign SVs have transmission rate being 0.64 (0.5 cutoff, binomial test p-value = 0.04) and 0.68 (quantile cutoff, binomial test p-value = 0.07), respectively. Consistent with our original analysis, we observed a slightly larger effect size of over-transmission pattern for paternally inherited pathogenic SVs than benign SVs. Due to the small sample sizes, no statistical significance was achieved when comparing transmission rate between predicted paternal pathogenic SVs and predicted benign paternal SVs (0.5 cutoff: two-sided proportion test: p-value=0.656; quantile cutoff: p-value=0.910).

Potential ascertainment biases between the test dataset of small and large SVs

As shown in **Figure 3b-c**, all models except for CADD-SV yielded lower AUCs in the test set of large SVs than those in the test set of small SVs. This finding aligns with the results presented in the StrVCTVRE[2] paper, where the authors reported higher AUCs for SVs categorized as either small(<30kb) or large (>500kb) than those with mid-length (30kb-500kb). The decreased model performance for larger SVs could be attributed to potential ascertainment biases between the test dataset of small SVs and the test dataset of large SVs, due to the differences in SV detection technologies. Specifically, larger SVs with sizes over 100kbp are primarily detected by microarrays (with imprecise breakpoints) and are more likely to be reported in literature. On the other hand, smaller SVs, ranging from 50bp to 100kbp, are commonly identified using Next-Generation Sequencing (NGS) techniques; most of these SVs are not reported in literature or documented in databases unless there is clear evidence for pathogenicity.

Supplementary tables and figures

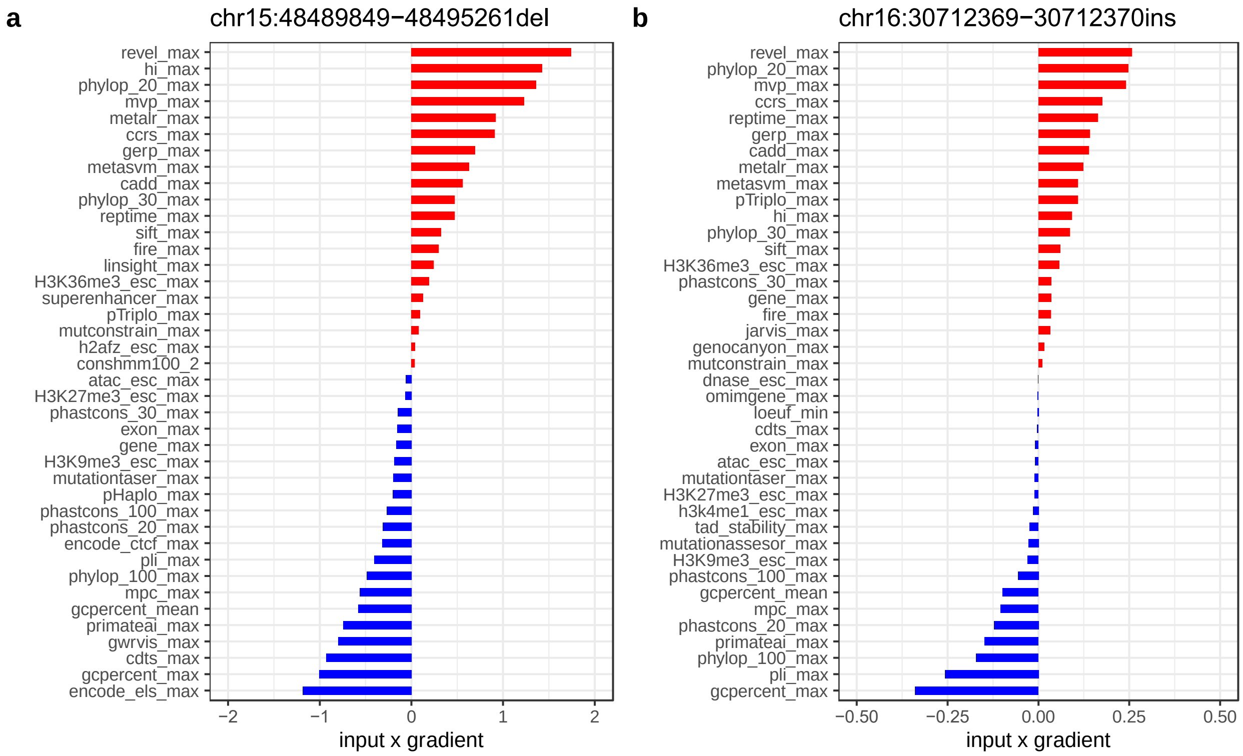

Figure S1. Feature importance for two novel SVs measured by input x gradient. Displayed are 20 features (y-axis) with largest (positive, red) input x gradient values (x-axis) and 20 features with smallest (negative, blue) input x gradient values (x-axis). Features with positive input x gradient values are ones driving p_{sv} to 1. Features with negative input x gradient values are ones driving p_{sv} to 0. **(a)** deletion at chr15:48452562-48463240, GRCh38 **(b)** insertion at chr16:30712369-30712370, GRCh38

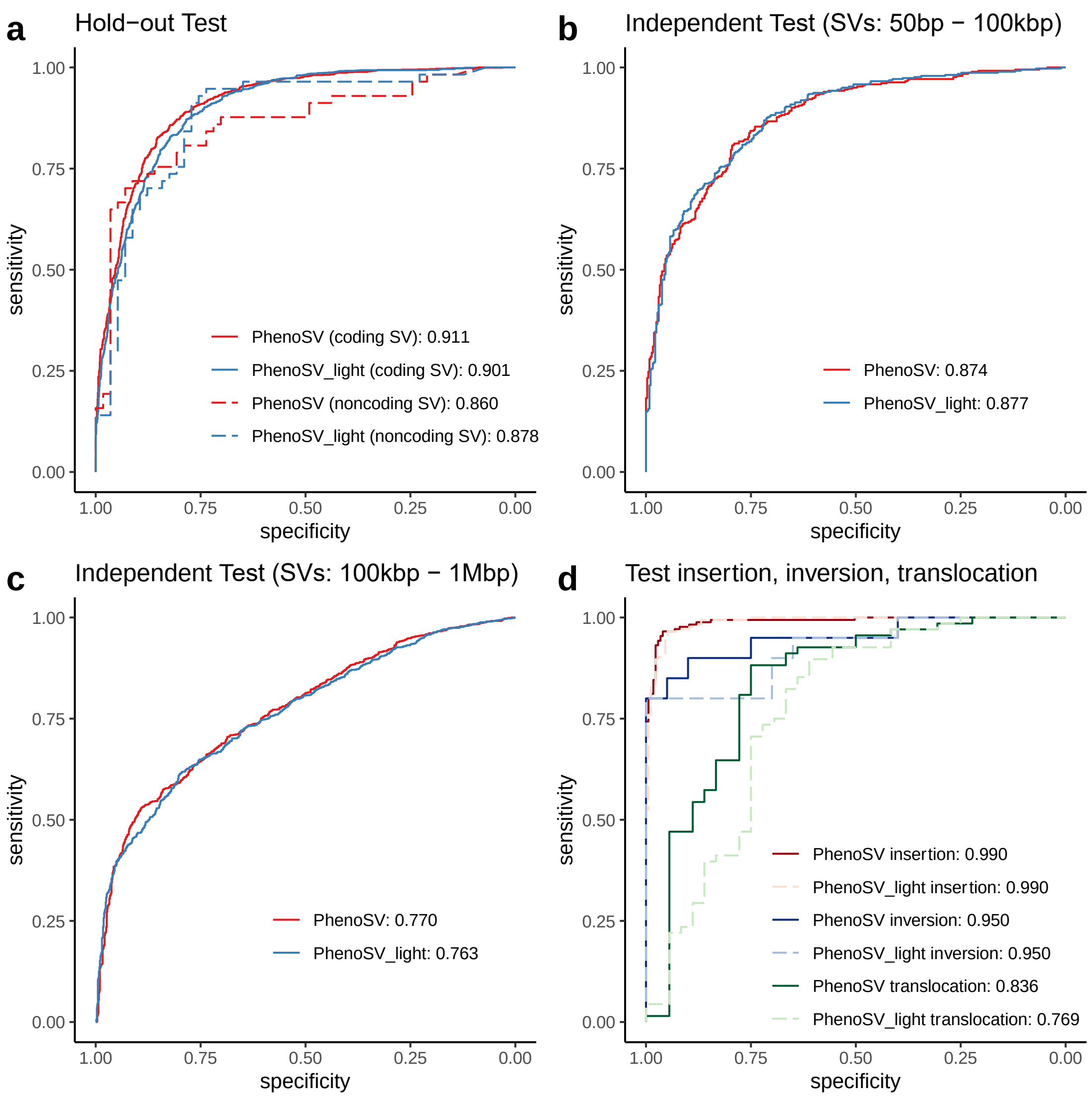

Figure S2. Comparison of model performance between PhenoSV (238 features) and PhenoSV-light (42 features). **(a)** Model AUCs in the hold-out test dataset for coding SVs (n=1,385 pathogenic and n=1,174 benign SVs, solid lines) and noncoding SVs (n=57 pathogenic and n=57 benign SVs, dashed lines). **(b-c)** Model AUCs in the independent test datasets of small coding SVs (n=383 pathogenic and n=366 benign SVs) with sizes ranging from 50bp to 100kbp and large coding SVs (n=1,208 pathogenic and n=801 benign SVs) with sizes ranging from 100kbp to 1Mbp. **(d)** Model AUCs in the test datasets of insertions (n=175 pathogenic SVs and n=175 benign SVs), inversions (n=20 pathogenic SVs and n=20 benign SVs), and translocations (n=68 pathogenic fusion transcripts and n=38 benign fusion transcripts). Source data are provided as a Source Data File.

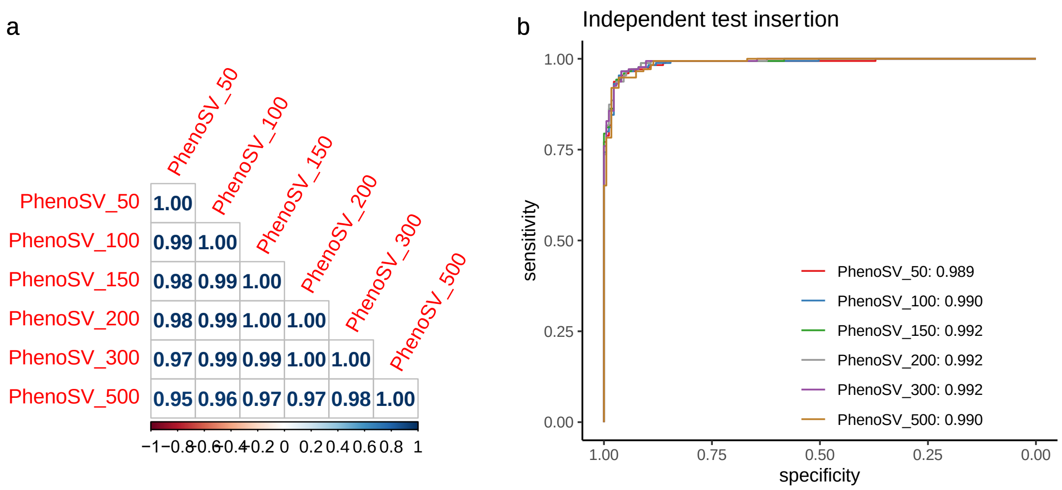

Figure S3. Impacts of window size on PhenoSV predictions. **(a)** Displayed are Pearson's correlation coefficients between PhenoSV_size1 predictions and PhenoSV_size2 predictions. **(b)** Model AUCs. Window sizes were set as 50bp, 100bp, 150bp, 200bp, 300bp, and 500bp. Test dataset of insertions (n=175 pathogenic SVs and n=175 benign SVs) was used for evaluation. Source data are provided as a Source Data File.

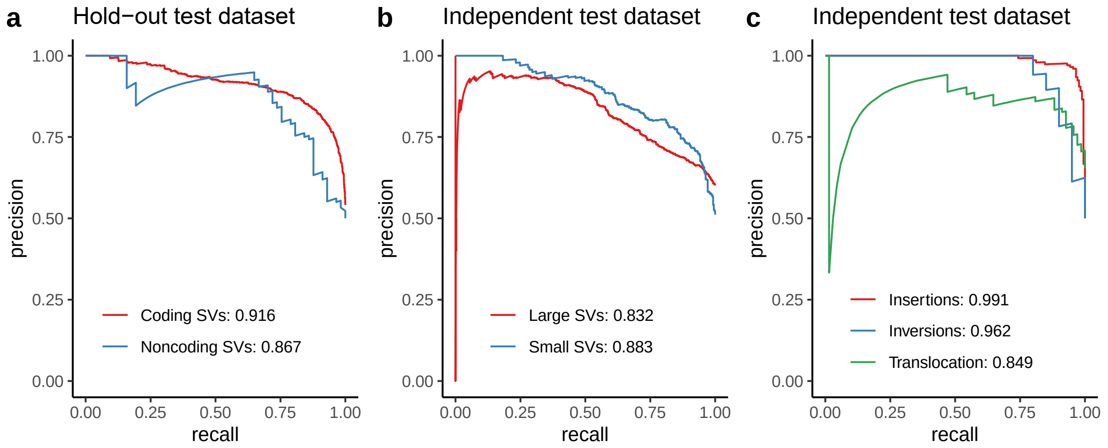

Figure S4. Precision-recall curves for PhenoSV. **(a)** Model auPRCs in the hold-out test dataset for coding SVs (n=1,385 pathogenic and n=1,174 benign SVs) and noncoding SVs (n=57 pathogenic and n=57 benign SVs). **(b)** Model auPRCs in the independent test datasets of small coding SVs (n=383 pathogenic and n=366 benign SVs) with sizes ranging from 50bp to 100kbp and large coding SVs (n=1,208 pathogenic and n=801 benign SVs) with sizes ranging from 100kbp to 1Mbp. **(c)** Model auPRCs in the test datasets of insertions (n=175 pathogenic SVs and n=175 benign SVs), inversions (n=20 pathogenic SVs and n=20 benign SV), and translocations (n=68 pathogenic fusion transcripts and n=38 benign fusion transcripts). Source data are provided as a Source Data File.

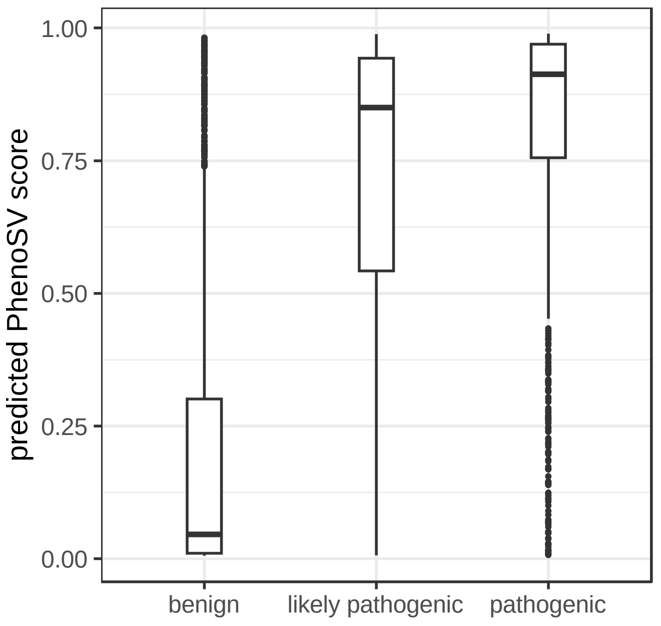

Figure S5. Boxplots of predicted PhenoSV score distributions in the hold-out test dataset for coding SVs from ClinVar (n=1,109 pathogenic SVs, n=276 likely pathogenic SVs, and n=1,174 benign SVs). Median (center line), IQR (box limits), and outliers (points) that exceeding 1.5x IQR were shown in the boxplot.

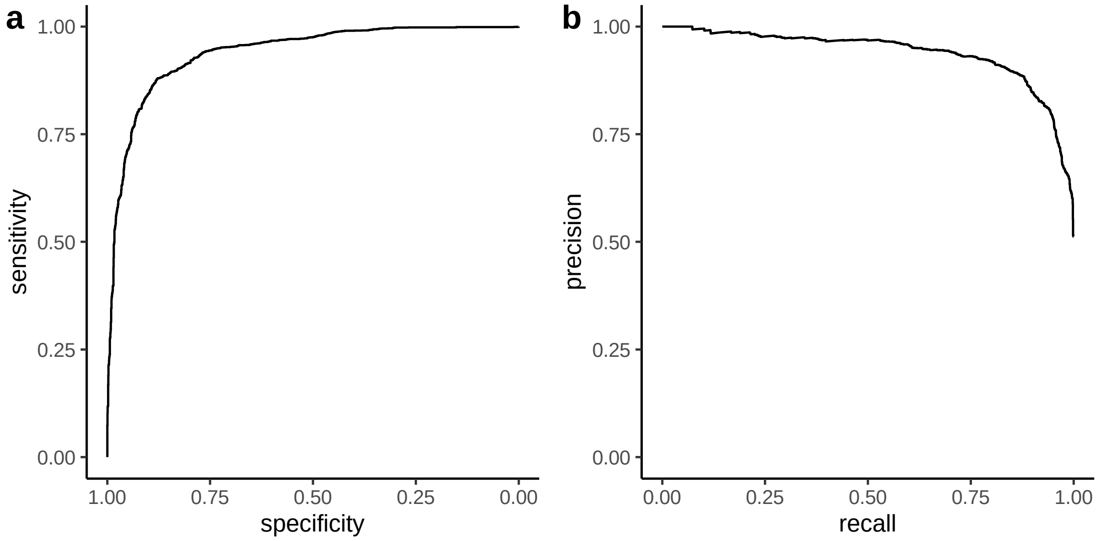

Figure S6. PhenoSV performance for sex chromosomes of chrX and chrY. **(a)** model AUC (0.94, 95% CI: 0.93-0.95) **(b)** model auPRC (0.94). PhenoSV performance was evaluated in the sex chromosome test dataset (2,034 pathogenic and 1,934 benign SVs). Source data are provided in the Source Data File.

Table S1. Model performance before and after calibrating PhenoSV scores

Dataset	Before Calibration				After Calibration			
	AUC	ACC	SEN	SPE	AUC	ACC	SEN	SPE
Hold-out test Coding (1385+, 1174-)	0.911	0.839	0.852	0.825	0.911	0.842	0.857	0.824
Hold-out test Noncoding (57+, 57-)	0.86	0.658	0.93	0.386	0.86	0.816	0.719	0.912
Independent test, Coding Small (383+, 366-)	0.874	0.777	0.731	0.825	0.874	0.776	0.731	0.822
Independent test, Coding Large (1208+, 801-)	0.77	0.667	0.514	0.899	0.77	0.67	0.519	0.898
Test insertion (175+, 175-)	0.99	0.937	0.989	0.886	0.99	0.937	0.983	0.891
Test inversion (20+, 20-)	0.898	0.875	0.8	0.95	0.95	0.875	0.75	1
Test translocation (68+, 38-)	0.836	0.731	1	0.222	0.836	0.731	1	0.222

Table S2. Counts of COSMIC CNVs that have overlapping genomic regions with rCNVs and corresponding HPO terms

Tumor Type	HPO	SV Number
bone osteosarcoma	HP:0002669	34
breast carcinoma	HP:0003002	428
central nervous system glioma	HP:0009733	15
central nervous system medulloblastoma	HP:0002885	24
large intestine carcinoma	HP:0100834	8
liver carcinoma	HP:0002896	178
lung carcinoma	HP:0100526	21
pancreas carcinoma	HP:0002894	454
prostate carcinoma	HP:0012125	1661
endometrium carcinoma	HP:0012114	17
skin malignant melanoma	HP:0002861	7
Total		2847

Table S3. Overlapped rCNV segments and COSMIC CNVs affecting three types of genes, including those associated with inherited diseases, those associated with cancers and those associated with both inherited diseases and cancers.

rCNV	rCNV ID	# of COSMIC CNVs (coding)	# of COSMIC CNVs (noncoding)
16p11.2	merged_DEL_segment_16p11.2_A; merged_DUP_segment_16p11.2_A	0	5
17p11.2	merged_DUP_segment_17p11.2; merged_DEL_segment_17p11.2	8	43
17q11.2	merged_DEL_segment_17q11.2	10	55
18p11.23-p11.32	merged_DUP_segment_18p11.23-p11.32	2	0
Total		20	103

Table S4. Comparision between PhenoSV and SvAnna in prioritizing phenotype-associated SVs.

Dataset	PhenoSV	SvAnna
Coding SVs, hold out test set (n=1,007)	83.81%	97.12%
Coding SVs, independent test set (n=494)	50%	20.64%
Noncoding SVs (n=193)	22.28%	8.29%
Insertions and inversions (n=149)	96.76%	87.92%

Values in the table are the percentage of simulated SV profiles whose true pathogenic SVs are prioritized within top 20 among ~19000 SVs

Table S5. Features in PhenoSV-light model

gcpercent_mean	remap_crm_max
cadd_max	utr5_max
hi_max	lncrna_max
pli_max	encode_els_max
pHaplo_max	loeuf_min
omimgene_max	gene_sum
metalr_max	superenhancer_sum
mvp_max	tad_stability_sum
mpc_max	chromhmm_8
ccrs_max	chromhmm_14
revel_max	conshmm_50_7
cdts_max	conshmm_50_32
gwrvis_max	conshmm_50_49
atac_esc_max	conshmm_100_28
dnase_esc_max	conshmm_100_38
h2afz_esc_max	conshmm_100_41
h3k4me3_esc_max	conshmm_100_47
H3K9me3_esc_max	conshmm_100_52
H3K27me3_esc_max	conshmm_100_86
H3K36me3_esc_max	conshmm_100_87
POLR2A_esc_max	SV type

Table S6. Model performance of splitting by chromosomes and splitting by random

Dataset	Split by chromosomes				Split by random			
	AUC	ACC	SEN	SPE	AUC	ACC	SEN	SPE
Hold-out test Coding (1385+, 1174-)	0.911	0.842	0.857	0.824	0.948	0.871	0.909	0.827
Hold-out test Noncoding (57+, 57-)	0.86	0.816	0.719	0.912	0.89	0.781	0.667	0.895
Independent test, Coding Small (383+, 366-)	0.874	0.776	0.731	0.822	0.876	0.796	0.791	0.801
Independent test, Coding Large (1208+, 801-)	0.77	0.67	0.519	0.898	0.769	0.669	0.54	0.863

Table S7. Transmission analysis with pathogenicity threshold of 0.5.

	PhenoSV stratification	paternal total	father transmitted	father untransmitted	father transmitted rate	Two-sided binomial test of father p	maternal total	mother transmitted	mother untransmitted	mother transmitted rate	Two-sided binomial test of mother p
**case**	all	100	67	33	0.67 (0.57, 0.76)	0. 0008737	79	47	32	0.59 (0.48, 0.70)	0.1147
	predicted pathogenic	41	29	12	0.71 (0.54, 0.84)	0.01151	47	28	19	0.60 (0.44, 0.74)	0.243
	predicted benign	59	38	21	0.64 (0.51, 0.76)	0.03634	32	19	13	0.59 (0.41, 0.76)	0.3771
**control**	all	26	16	10	0.62 (0.41, 0.80)	0.3269	17	10	7	0.59 (0.33, 0.82)	0.6291
	predicted pathogenic	11	7	4	0.64 (0.31, 0.89)	0.5488	13	8	5	0.62 (0.32, 0.86)	0.5811
	predicted benign	15	9	6	0.60 (0.32, 0.84)	0.6072	4	2	2	0.50 (0.07, 0.93)	1

Table S8. Transmission analysis with pathogenicity threshold of 30% and 70% quantiles.

	PhenoSV stratification	paternal total	father transmitted	father untransmitted	father transmitted rate	Two-sided binomial test of father p	maternal total	mother transmitted	mother untransmitted	mother transmitted rate	Two-sided binomial test of mother p
**case**	all	100	67	33	0.67 (0.57, 0.76)	0.0008737	79	47	32	0.59 (0.48, 0.70)	0.1147
	predicted pathogenic	29	21	8	0.72 (0.53, 0.87)	0.02412	24	11	13	0.46 (0.25, 0.67)	0.8388
	predicted benign	31	21	10	0.68 (0.49, 0.83)	0.07076	25	12	13	0.48 (0.28, 0.69)	1
**control**	all	26	16	10	0.62 (0.41, 0.80)	0.3269	17	10	7	0.59 (0.33, 0.82)	0.6291
	predicted pathogenic	9	6	3	0.67 (0.30, 0.93)	0.5078	5	2	3	0.4 (0.05, 0.85)	1
	predicted benign	7	4	3	0.57 (0.18, 0.90)	1	4	2	2	0.5 (0.07, 0.93)	1

Supplementary Reference:

1 de Cid, R. *et al.* Deletion of the late cornified envelope LCE3B and LCE3C genes as a susceptibility factor for psoriasis. *Nat Genet* **41**, 211-215 (2009). https://doi.org/10.1038/ng.313

2 Sharo, A. G., Hu, Z., Sunyaev, S. R. & Brenner, S. E. StrVCTVRE: A supervised learning method to predict the pathogenicity of human genome structural variants. *Am J Hum Genet* **109**, 195-209 (2022). https://doi.org/10.1016/j.ajhg.2021.12.007
