# Supplementary material for: PhenoSV: interpretable phenotype-aware model for the prioritization of genes affected by structural variants
Source: Nat Commun. 2023 Nov 28;14:7805. doi: 10.1038/s41467-023-43651-y (PMC10684511; doi:10.1038/s41467-023-43651-y)
Supplement: Supplementary file 3 — Description of Additional Supplementary Files [file 41467_2023_43651_MOESM3_ESM.pdf]

Description of Additional Supplementary Files:

Supplementary Data 1: Genomic features used to annotate SVs

Supplementary Data 2: Summary of datasets used in the study
