# Supplementary material for: PhenoSV: interpretable phenotype-aware model for the prioritization of genes affected by structural variants
Source: Nat Commun. 2023 Nov 28;14:7805. doi: 10.1038/s41467-023-43651-y (PMC10684511; doi:10.1038/s41467-023-43651-y)
Supplement: Supplementary file 5 — Reporting Summary [file 41467_2023_43651_MOESM5_ESM.pdf]

nature portfolio

Corresponding author(s): Kai Wang

Last updated by author(s): Oct 20, 2023

Reporting Summary

Nature Portfolio wishes to improve the reproducibility of the work that we publish. This form provides structure for consistency and transparency in reporting. For further information on Nature Portfolio policies, see our Editorial Policies and the Editorial Policy Checklist.

Statistics

For all statistical analyses, confirm that the following items are present in the figure legend, table legend, main text, or Methods section.

n/a	Confirmed	
☐	☒	The exact sample size (n) for each experimental group/condition, given as a discrete number and unit of measurement
☒	☐	A statement on whether measurements were taken from distinct samples or whether the same sample was measured repeatedly
☐	☒	The statistical test(s) used AND whether they are one- or two-sided<br>*Only common tests should be described solely by name; describe more complex techniques in the Methods section.*
☒	☐	A description of all covariates tested
☐	☒	A description of any assumptions or corrections, such as tests of normality and adjustment for multiple comparisons
☐	☒	A full description of the statistical parameters including central tendency (e.g. means) or other basic estimates (e.g. regression coefficient) AND variation (e.g. standard deviation) or associated estimates of uncertainty (e.g. confidence intervals)
☐	☒	For null hypothesis testing, the test statistic (e.g. F, t, r) with confidence intervals, effect sizes, degrees of freedom and P value noted<br>*Give P values as exact values whenever suitable.*
☒	☐	For Bayesian analysis, information on the choice of priors and Markov chain Monte Carlo settings
☒	☐	For hierarchical and complex designs, identification of the appropriate level for tests and full reporting of outcomes
☐	☒	Estimates of effect sizes (e.g. Cohen's d, Pearson's r), indicating how they were calculated

Our web collection on statistics for biologists contains articles on many of the points above.

Software and code

Policy information about availability of computer code

Data collection	No software was used for data collection.
Data analysis	The manuscripts develops a new software tool, which is available at https://github.com/WGLab/PhenoSV. A companion web server can be accessed at https://phenosv.wglab.org. PhenoSV was trained using Pytorch Lightning framework (1.6.4). R package of caret (version 6.0-94) was used for tunning XGBoost hyperparameters, and XGBoost model was trained using R package of xgboost (version 1.7.5.1). Predictions of benchmark methods of CADD-SV, AnnotSV, and StrVCTVRE were obtained from the official web servers by uploading bed files containing test SV coordinates (CADD-SV: https://cadd-sv.bihealth.org; AnnotSV: https://lbgi.fr/AnnotSV/; StrVCTVRE: https://strvctvre.berkeley.edu). Predictions of SVScore (https://github.com/lganel/SVScore) were obtained by running the command line tool. SvAnna (https://github.com/TheJacksonLaboratory/SvAnna) was used to benchmark SV prioritization performance. All statistical analyses were performed in R, version 4.2.2. AUC values for model performances were calculated using R package of pROC (1.18.4).

For manuscripts utilizing custom algorithms or software that are central to the research but not yet described in published literature, software must be made available to editors and reviewers. We strongly encourage code deposition in a community repository (e.g. GitHub). See the Nature Portfolio guidelines for submitting code & software for further information.

Data

Policy information about availability of data

All manuscripts must include a data availability statement. This statement should provide the following information, where applicable:

- Accession codes, unique identifiers, or web links for publicly available datasets
- A description of any restrictions on data availability
- For clinical datasets or third party data, please ensure that the statement adheres to our policy

The simulation and benchmarking data are provided at https://github.com/WGLab/PhenoSV. The training and testing data can be accessed through https://www.ncbi.nlm.nih.gov/clinvar/ (ClinVar, full release 02/2022), https://www.deciphergenomics.org (DECIPHER, v11.15), https://cancer.sanger.ac.uk/cosmic (COSMIC, release v97 for fusion gene data and release v96 for somatic CNV data), https://www.ncbi.nlm.nih.gov/dbvar/ (dbVar: nstd186, nstd152, nstd162, nstd175), and supplementary files of according papers mentioned in methods section.

Research involving human participants, their data, or biological material

Policy information about studies with human participants or human data. See also policy information about sex, gender (identity/presentation), and sexual orientation and race, ethnicity and racism.

Reporting on sex and gender	N/A
Reporting on race, ethnicity, or other socially relevant groupings	N/A
Population characteristics	N/A
Recruitment	N/A
Ethics oversight	N/A

Note that full information on the approval of the study protocol must also be provided in the manuscript.

Field-specific reporting

Please select the one below that is the best fit for your research. If you are not sure, read the appropriate sections before making your selection.

[x] Life sciences [ ] Behavioural & social sciences [ ] Ecological, evolutionary & environmental sciences

For a reference copy of the document with all sections, see nature.com/documents/nr-reporting-summary-flat.pdf

Life sciences study design

All studies must disclose on these points even when the disclosure is negative.

Sample size	The training dataset contains 14,622 SVs with 14,292 being coding SVs and 330 being noncoding SVs. The validation dataset contains 2,182 SVs, and the hold-out test dataset contains 2,673 SVs. The sample size was determined by the number of all available SV samples in the databases mentioned in the manuscript.
Data exclusions	SVs were excluded from training dataset if they do not fulfill all the following requirements: (1) clinical significance of benign, benign/likely benign, likely pathogenic, pathogenic, pathogenic/likely pathogenic; (2) not somatic in origin; (3) type of copy number loss, deletion, copy number gain, duplication, insertion, or inversion; (4) SV size ranges from 50bp to 1Mbp; (5) best placement in the presence of multiple placements per assembly; (6) autosomal.
Replication	This study does not involve any trials in study design, thus not applicable for replication
Randomization	This study does not involve any trials in study design, thus not applicable for randomization
Blinding	This study does not involve any trials in study design, thus not applicable for blinding.

Reporting for specific materials, systems and methods

We require information from authors about some types of materials, experimental systems and methods used in many studies. Here, indicate whether each material, system or method listed is relevant to your study. If you are not sure if a list item applies to your research, read the appropriate section before selecting a response.

Materials & experimental systems

n/a	Involved in the study
☒	☐ Antibodies
☒	☐ Eukaryotic cell lines
☒	☐ Palaeontology and archaeology
☒	☐ Animals and other organisms
☒	☐ Clinical data
☒	☐ Dual use research of concern
☒	☐ Plants

Methods

n/a	Involved in the study
☒	☐ ChIP-seq
☒	☐ Flow cytometry
☒	☐ MRI-based neuroimaging
